# Developing a framework for successful research partnerships in global health

**DOI:** 10.1186/s12992-016-0152-1

**Published:** 2016-05-06

**Authors:** Fiona Larkan, Ogenna Uduma, Saheed Akinmayọwa Lawal, Bianca van Bavel

**Affiliations:** Centre for Global Health, Trinity College Dublin, Dublin, Ireland; Department of Sociology/Psychology, OlabisiOnabanjo University, Ago-Iwoye, Ogun State Nigeria

**Keywords:** Guidelines for academic partnerships, Relational, Operational, Core concepts, Research framework

## Abstract

**Background:**

The Centre for Global Health, Trinity College Dublin has as one of its goals, strengthening health systems in developing countries. In realising this goal we work across more than 40 countries with third-level, civil society, government, private sector and UN partners. Each of these requires that different relationships be established. Good principles must guide all global health research partnerships.

An exploratory research project was undertaken with research partners of, and staff within, the Centre for Global Health. The aim was to build an evidence-based framework.

**Methods:**

An inductive exploratory research process was undertaken using a grounded theory approach in three consecutive phases: Phase I: An open-ended questionnaire was sent via email to all identified partners. Phase II: A series of consultative meetings were held with the staff of the Centre for Global Health. Phase III: Data sets from Phases I and II were applied to the development of a unifying framework. Data was analysed using grounded theory three stage thematic analysis - open, axial and selective coding.

**Results:**

Relational and operational aspects of partnership were highlighted as being relevant across every partnership. Seven equally important core concepts emerged (focus, values, equity, benefit, leadership, communication and resolution), and are described and discussed here. Of these, two (leadership and resolution) are less often considered in existing literature on partnerships.

**Conclusions:**

Large complex partnerships can work well if all parties are agreed in advance to a common minimum programme, have been involved from the design stage, and have adequate resources specifically allocated. Based on this research, a framework for partnerships has been developed and is shared.

**Electronic supplementary material:**

The online version of this article (doi:10.1186/s12992-016-0152-1) contains supplementary material, which is available to authorized users.

## Background

Health research is an influential component of improving health outcomes, distributing knowledge, improving technological effectiveness and addressing the conundrum of inequitable health care [1]. In order to advance health research and increase health-systems capacity, it is necessary to develop multi-country collaborative health research networks [2]. Collaborations in global health research, however, while tremendously advantageous, can be demanding and challenging; many partnerships struggle to make the most of collaborative processes to accomplish their stated goals [3]. Capacity strengthening, in terms of global health research, needs to be seen as more than simply the transfer of knowledge, skills and resources, but the ‘global and collective struggle for health and well-being in which we are partners’ [4], (Page 3). This paper seeks to stimulate insight, discussion and future research to inform successful partnership practice in global health research.

### Definition

For the purposes of this paper, we define health partnerships as “contextually relevant peer-to-peer collaborations which offer a platform for sharing knowledge and growing expertise globally, working towards a common goal, across disciplines and perspectives”. This admittedly broad definition of partnership is cognisant of the multiple type of partnerships in global health research which exist on a broad spectrum to address health inequalities [5], improve health care delivery, evaluate health programmes and interventions [6], develop collaborative approaches to international research [7], build capacity for health researchers [8], strengthen public health education [9], examine health promotion through international collaboration [10], rethink health research capacity strengthening [4], sustain social networks for global health research [11], and to relate inter-organisational and network learning to education partnerships [12, 13], amongst other prominent issues in the discourse of research partnership in global health. A broad definition such as this encompasses the key attributes which form all such partnerships and enables success, and in that sense can be seen as unifying. Hence we consider the framework itself as having the capacity to be a unifying framework.

However, it must be acknowledged that part of the challenge of framing this discussion has come from the diversity within the partnership literature itself, given the myriad of disciplines, perspectives, contexts, and practical applications, knowledge and experiences of partners. The partnership framework we propose here emerges out of primary research and offers a perspective on the core issues as concepts (focus, values, equity, benefit, communication, leadership and resolution) that must be considered when engaging partnership for global health research. Moreover, the framework shows the process most global health partnerships pass through - from their formation, to their implementation, monitoring and evaluation, and, where appropriate, their dissolution - in order to achieve success. The flexibility of this framework is intended to guide politically and socially contextualised [7], successful research partnerships in global health, along with an appreciation of the cultural realities in which they operate, in order to achieve the desired goals and objectives.

### Centre for Global Health, Trinity College Dublin

As an international academic research institution, the Centre for Global Health (CGH), Trinity College Dublin, promotes the collaboration of partnerships towards the realisation of strengthening health systems in lower-middle income-countries (LMIC). We recognise that the functionality of different partnerships in different contexts requires different input which will inevitably produce a diversity of outcomes. Since its establishment in 2005, the CGH has worked across more than 40 countries with an international network of more than 20 third-level academic, civil society, government, private sector and UN partners.

Within this period CGH has engaged in inter-institutional and intra-institutional forms of partnerships with the aim of improving health systems performance. Partnerships have include those focused on capacity-building, implementation research, health policy and systems research, participatory action research, impact orientation, interdisciplinary approaches, and long-term partnerships for health systems strengthening in the global south [5]. One example of the kind of partnership undertaken in recent years is the international doctorate in global health programme (Indigo); a north-south collaboration aimed at building the capacity of southern partner universities through extensive training of selected junior staff. Partners collectively deliver the interdisciplinary international doctorate, focusing on social health science and strengthening health systems. Programme partners are TCD (Ireland), the Mailman School of Public Health at Columbia University, New York City (USA), Harvard School of Public Health, Boston (USA), the Methodology Hub at Queen’s University, Belfast (Northern Ireland), and the Cochrane Centre in Oxford (UK), along with four universities in sub-Saharan Africa: Addis Ababa University (Ethiopia), University of Ibadan (Nigeria), Makerere University (Uganda) and University of Malawi College of Medicine (Malawi). The programme also works collaboratively with the Human Sciences Research Council in South Africa and the Council on Health Research for Development based in Geneva, Switzerland. The Indigo programme emerged from ongoing debates around aid effectiveness, academic collaboration between universities and institutions in low- and middle -income countries and, more specifically, the widely recognised need to strengthen health system research in Africa [14]. A table of selected additional CGH partnerships is shown in Appendix 1.

The ten year anniversary of the establishment of CGH provided an opportune moment to reflect on, and (re)evaluate, the course of these partnerships. To this end we conducted exploratory research involving current and former partners and staff members, which sought to identify the characteristics of successful research partnerships and to reflect on some of the lessons learned. The results of this research informed the development of a unifying framework for establishing successful research partnerships in global health. This framework, and the process through which it was developed, is presented below.

#### Literature

After a decade of building relationships through a range of partners and operations, practice and research, the CGH recognises the importance of guiding principles. Several useful sets of guidelines have been established (in particular, for example, the Swiss Commission for Research Partnership with Developing Countries (KFPE) principles [15, 16], and the Netherlands Development Assistance Research Council (RAWOO) guidelines [17]). In response to the inequitable exclusion of Southern partners and voices contributing to written best practices for North-South health research partnerships, the Canadian Coalition for Global Health Research (CCGHR) in collaboration with the International Development Research Centre (IDRC), BRAC (Bangladesh), the Universidad Andina Simon Bolivar (Ecuador), and the Armauer Hansen Research Institute (Ethiopia) developed a Partnership Assessment Tool (PAT) that can be used to improve the ethical conduct and accountability of partners [9]. Helping to navigate questions about authorship and intellectual property as well as discussion points on the development of MOUs, and priorities, PAT’s application is seen through four phases of partnering: inception, implementation, dissemination, and good endings with new beginnings [18]. The CCGHR is currently leading a participatory research process in developing principles for guiding involvement in equitable global health research [19]. More recently, the Council on Health Research for Development (COHRED) released a ‘Fairness Index’ for international collaborative partnerships, an extension of its Fair Research Contracting (FRC) initiative for better global impact [20]. The index provides a certification mechanism for partners engaging in research to encourage and improve upon best-practices within their international collaborations; including the alignment of interests, long term contributions and reduction of inequity to stimulate socio-economic development.

While existing evaluations and studies on Global South-North partnerships, endeavours and networks engaging in academic research, have been instructive and evidence informed, many of the issues that have been recognised warrant further exploration and action [21]. The formation of global health research partnerships requires conceptual explanation to inform future practice and explore the resolution of identified issues.

Neither global health research, nor global health research partnerships, are removed from underlying political inequalities, competition, economic growth or opportunity [22]. Similarly, social, cultural, environmental, and technological realities exist which shape and define successful research partnerships in global health. Understanding these factors will direct and guide partnership formation, and ensure the achievement of desired goals.

While the advancements and benefits to health research arising from global partnerships are undeniable, there are persisting issues around inequality and distribution of these benefits between Northern and Southern partners—a ‘hierarchy of research and research outputs’ [22–24]. Numerous grants and funding regimes incentivise partnerships between high-income countries (HICs) and LMICs, and it is important that donors recognise, and are sensitive towards, the disparity of existing and contributing capacities within this international market. In addition, donor-funded programmes often have a shorter lifespan than that necessary to achieve long-term mutually beneficial and sustaining partnerships [25]. Finding a balance between merit and equitable funding allocation would help to maintain successful working relations and garner the necessary buy-in towards the realization of goals and outcomes [25], ensuring that all partners benefit equitably.

Various organisational bodies and networks have begun to take action on some of the aforementioned issues, in particular the inequity of roles, responsibilities, power and benefits between Northern and Southern partners [26–28]. According to Costello & Zumla [29], while the extensive benefits of collaboration in global health research are recognised, there must be equitable balance between local and foreign partners to prioritise objectives and set research agendas in order for partnerships to be mutually beneficial. Their efforts in establishing four, cooperative principles of research partnerships applied to ‘developing countries’ come from a genuine desire for partnerships to promote positive change and were among some of the first to counter structural limits of persisting colonial systems. In recognition of the differing capacities, the costs, benefits and associated risks of partnering, Costello and Zumla [29] promote accountability of partners themselves, and even suggest that funding agencies need to play a more active role in monitoring the cooperation and equality of research partnerships. While they provide a checklist, it is evident the need for defined methods of applying such principles in the wider governance of partnerships [28]. In applying a clear mechanism for equitable partnering, they also refer to tokenistic and exploitative partnerships formed in sole fulfilment of funding and publication requirements. Such inequities are driven by structural power imbalances engrained within development, donor and research agendas, (Page 11/12). We suggest rather than simply monitoring, the donor-recipient relationship should be considered an equitable partnership, and be guided by this framework in the same way as research partners. In combination with these existing mechanisms, we see our contribution as the development of this unifying framework for successful global health research partnerships

## Methods

An inductive exploratory research process was undertaken with research partners of, and staff within, the Centre for Global Health. This research used a grounded theory approach in three consecutive phases [30]. The aim of the research was to build an evidence-informed framework for successful global health research partnerships. Ethical approval was granted by the HPM/CGH Research Ethics Committee at Trinity College Dublin (Ref. 11E/2015/05) and the principles of the Helsinki Declaration were complied with throughout.

### Phase I: Consultative process with partners

Partners involved in research collaborations across 22 institutions (universities, research institutes, non-governmental organizations and international organizations) mainly from social science and public health backgrounds were contacted via email and invited to complete a questionnaire consisting of six open-ended questions which sought to identify important features of successful partnerships (Additional file 1). 17 questionnaires were returned (9 from the south and 8 from the north) with a response rate of 77 %. The text generated from responses was thematically analysed independently by two researchers. Any discrepancies were discussed and resolved in a team meeting of all four authors. Responses were used to gather insight on the extent of each partner’s engagement, and their own understanding of how partnerships are created, what makes them successful, fair, and what (if any) is their value.

### Phase II: Consultative process with CGH staff members

A series of four consultative discussions was held with a core group of ten CGH staff members (8 from the north and 2 from the south), led by two members of the research team. During these meetings people were invited to share and exchange their own perspectives, experiences, and expectations on partnerships. Discussions were recorded with written notes which were fed back to participants after each meeting. These notes were transcribed and thematically analysed. Each round of consultation fed directly into the next series of discussions.

### Phase III: Development of a unifying framework

Analysis from the first two phases of this study became the focus of multiple research team discussions facilitated by one team member. We identified the underlying elements for developing a unifying framework to guide and inform the process of successful research partnerships in global health. A draft framework was presented at a seminar, after which comments and feedback were considered during the development of the final framework. Details of this process are outlined in the analysis below.

## Results

A total of seventeen partners completed and returned questionnaires. Of these nine were southern and eight northern-based partners, and included research institutions, civil society organisations, private companies and networks. Despite the deeply contextualised needs of each partner, our analysis of data from phase 1 and phase 2 found similar key themes necessary for successful partnerships.

Following a grounded theory approach, our first round open-coding resulted in a wide range of themes which we have designated as attributes [30]. Second round axial-coding, was conducted individually by two members of the team, and then by the full team, to condense these attributes into seven core concepts. These concepts feed directly into our framework. The final framework was developed based on the thematic analysis from the two phases of data collection with partners and staff members, as well as the referenced literature.

**Fig. 1:**
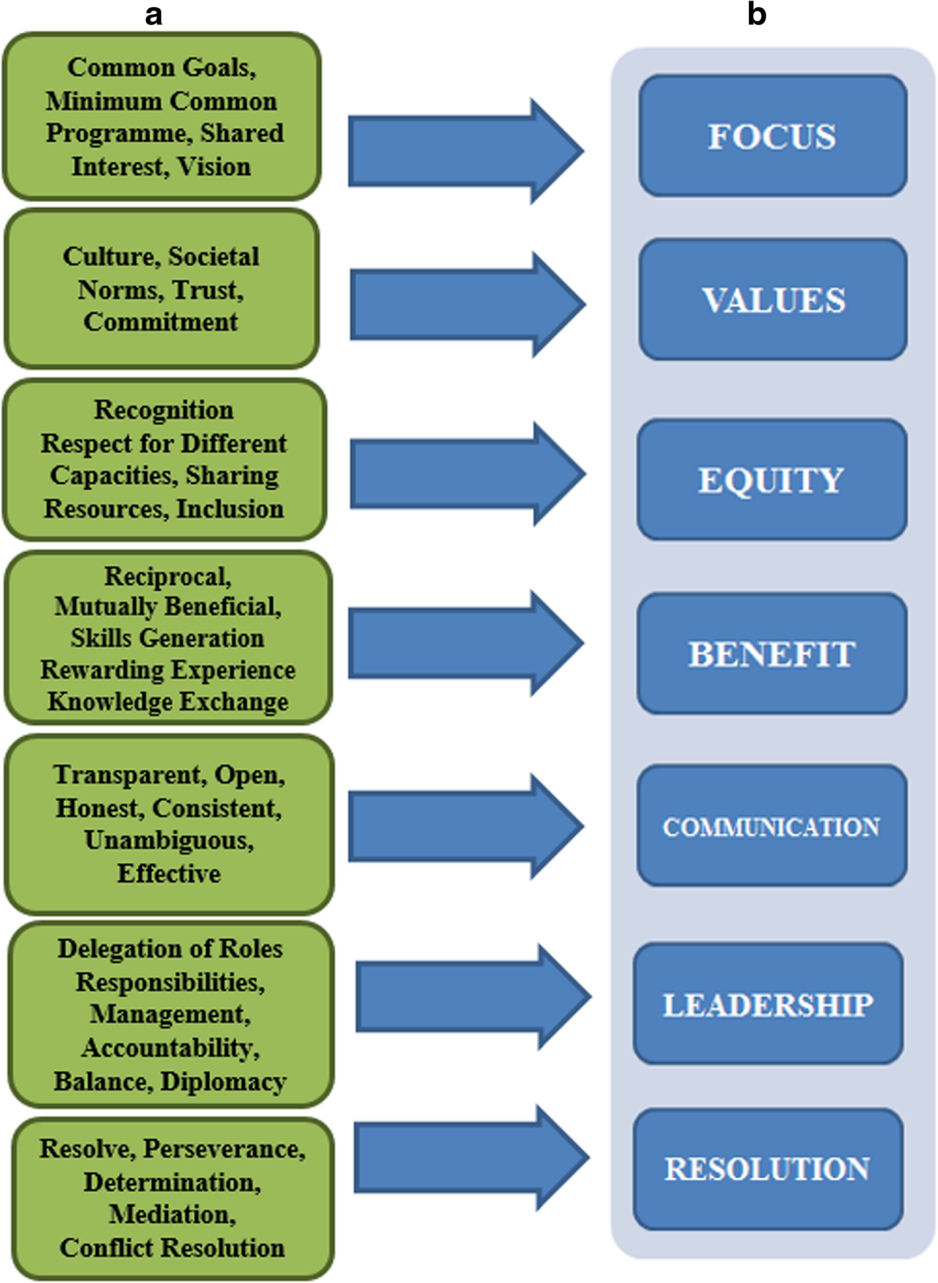
Consolidated sets of attributes (**a**) and derived core concepts (**b**) for successful research partnerships in Global Health

Based on the open coding thematic analysis a list of attributes was compiled for each of the core concepts. Examples and explanations of attributes are given below. While these attributes represent the views of our partners this is not an exhaustive list. However our review of the literature and early presentations of this research suggest that these attributes, listed as ‘A’ in Fig. 1, are desirable qualities in any partnership.

Seven core concepts emerged from a second level thematic analysis (axial coding). Listed ‘B’ in Fig. 1, these are Focus, Values, Equity, Benefit, Communication, Leadership, and Resolution. Figure 1 shows the derivation of core concepts from attributes.

Data suggests that while objectives are important, they are not in and of themselves sufficient to ensure focus. *Common goals* and *minimum common programme* among partners were identified as essential attributes. Similarly important, though perhaps more amorphous, *shared interest* and *vision* will help to keep partners focussed and motivated. Values in this context, refers to understanding the organisational *culture* of each partner and the underlying *societal norms* within which each partner operates. *Trust* was identified by all participants as a prerequisite, and a *commitment* to maintaining trust throughout as essential to successful partnership.

It is clear from our responses that equity rather than equality is a pillar of successful partnerships. This calls for *recognition* of, and *respect* for, differing *capacities*; and a *sharing of resources* such that *inclusion* occurs on an equitable basis. While the successful outcome of any global health research programme or project should accrue benefits to people/communities/organisations beyond the immediate partnership, benefit in the current context refers to *reciprocal* and *mutually beneficial* relationships among all partners. Such benefits may include the *generation of skills*, *rewarding experiences*, *knowledge exchange* etc.

Successful global health research partnerships, as reported by our participants, are dependent on *transparent*; *open*; *honest*; *consistent*; *unambiguous*, and *effective* communication. Leadership incorporates not only the *delegation* of *roles and responsibilities*, but also *management* and *accountability*. In particular *balance* and *diplomacy,* when dealing with all collaborators in the partnership, were identified as essential.

Resolution is a core concept that functions in two discrete ways. Firstly, there should be an acknowledgement that partnerships may encounter difficulties, and *resolve, perseverance*, and *determination* will be required to deal with any such difficulties. Secondly, while the on-going processes of *mediation* and *conflict resolution* may offer solutions, the need for the dissolution of partnerships may still ensue. As such results from this study indicate the need for partners to consider appropriate exit strategies during the partnership formation stage. Having a program or management mechanism specific to monitoring and revision processes will help determine the appropriate action and rectification of a situation, and ensure the best-possible outcome for all partners [31].

**Fig. 2:**
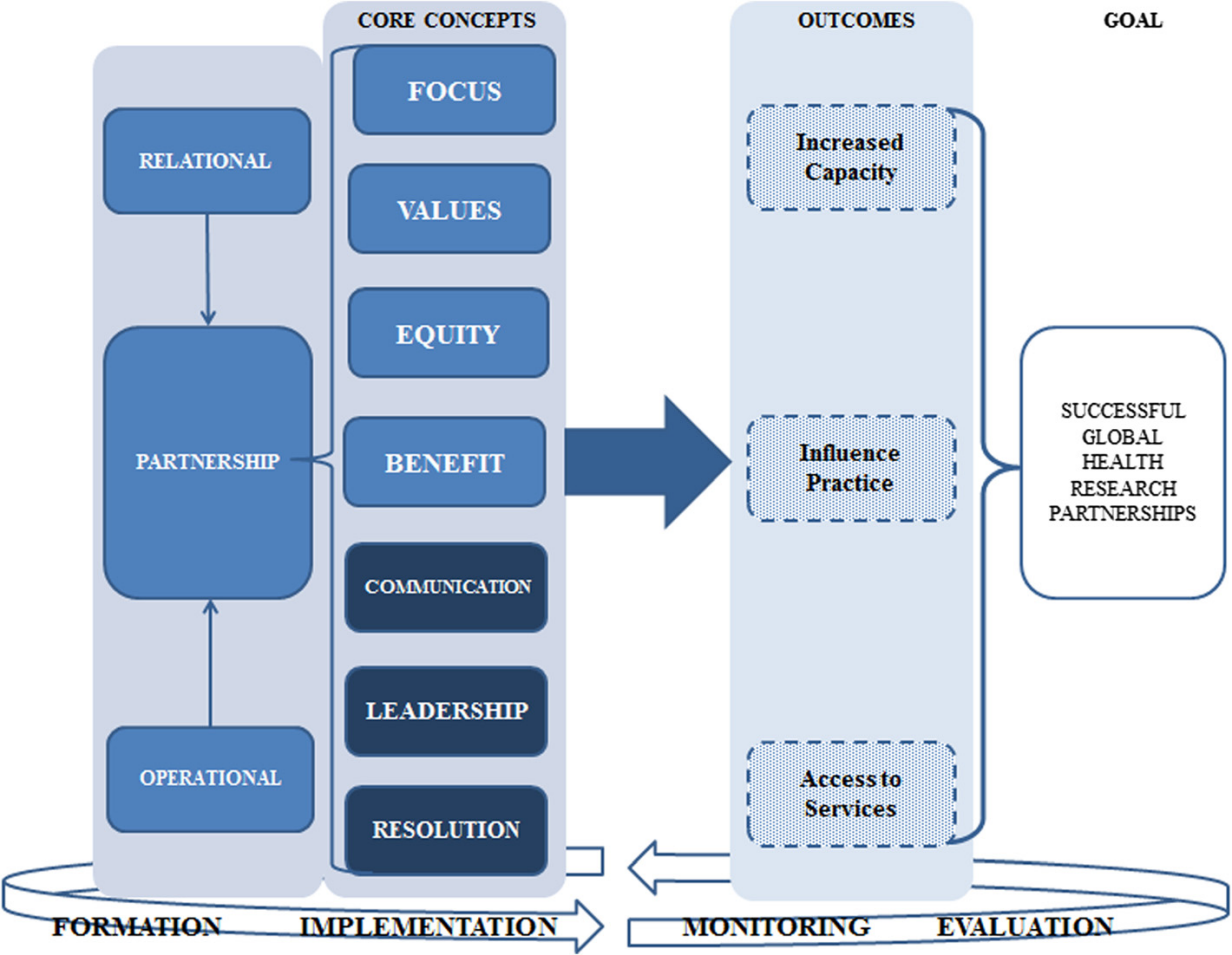
Framework for successful research partnerships in Global Health

Our third phase of thematic analysis applied a selective coding process to this data [30]. Emerging from this process we identified both relationships and operations as equally important. We have therefore incorporated both relational and operational aspects of partnerships in our framework (Fig. 2), as our data suggests that these underlie all seven of the core concepts. While there are overlaps amongst the attributes and their associated core concepts, it is important to note that no clear hierarchy was evident from the data. All seven concepts, therefore, should be considered as equally important pillars of successful global health research partnerships.

Our framework currently shows increased capacity, influenced practice, and influenced policy as examples of desirable outcomes in global health research. Outcomes, however, will be based on individual partnerships, their purpose, and their field of activities. Our data suggests that the application of these core concepts to the formation of desired outcomes will enhance the probability of successful global health research partnerships.

While we display the framework within a process for developing successful partnerships, this is not a linear process that can be abandoned once the partnership has been established. These core concepts need to be actively applied throughout the formation, implementation, monitoring, and evaluation of global health research partnerships.

## Discussion

The dominant discourse in global health research continues to negate the complexity of historical and contextual factors, particularly those at an institutional level, which arguably bear some of the greatest influence on the successful implementation of global health research initiatives, including the development of Southern-led research agendas, the re-organisation of funding streams, and culturally resonant evaluations and outputs [4, 27, 28]. These challenges, imposed and sustained by northern dominated perspective, are still relevant in the partnerships of today. Notwithstanding the present framework, existing principles and recommended guidelines considered, without further critical reflection, the extent of good intentions will continue to lead to negative consequences and unethical conduct.

Partnerships are often established around an activity or set of activities in which there are well defined common objectives and shared benefits, where each partner makes continuing contributions in one or more strategic areas [32]. Much of the focus in existing literature is on pragmatic outputs and productive outcomes of partnerships—what we have defined as *operations*—minimising the subjectivity of the interactional processes and underlying relationships. Our data suggests that global health partnerships are dependent upon successful relationships as much as (if not more than) successful operations; the two are inter-dependent.

Global health research involves multi-or bilateral actors which include (among others) multilateral organisations, governments, multinational and local corporations, non-governmental organisations (NGOs), community based organisations, universities and think tanks [33]. The level of global health partnerships can be local—local or local—global, and can be inter-institutional and intra-institutional collaborations. In all collaborations, while the attributes may vary, the core concepts remain the same. These core concepts (focus, values, equity, benefit, communication, leadership and resolution) and the attributes that contribute to them, as reported in this study are required for successful research partnership in global health. The need for a *common goal* among all partners engaged in any type of global health partnership is essential. Having a clear and common goal will ensure the project is achieved at the set time. Other attributes under Focus are seen in Fig. 1. Therefore for global health partnerships to succeed there is need for all partners to stay focussed on ensuring that their project objectives are realised. Having shared goals and aims, which are understood and accepted as being important by each partner, leads to improved coordination of policies, programs, and service delivery, and, ultimately, better outcomes [31], even in global health.

Values as a core concept that is built on *commitment, trust, culture and societal norms* as some of its key attributes is required for successful research partnerships in global health. Despite the challenges and risks of partnering, there is immense opportunity to be had when partners share similar values regarding research collaborations [27]. This is vital because the basis upon which the philosophy of any research partnership is constructed and established will thrive on the values of all partners involved. For example, trust has been identified as a critical factor that facilitates the development, effectiveness, and sustainability of community-academic partnerships [34]. Trust, as an attribute, is required for success in business and governance [36]. When translated into research partnerships in global health, individuals and institutions that have partnered with the CGH have all brought in trust into the partnerships formed which has been central to the eventual outcome of the projects done. Trust emerges in this study as a critical attribute and has been reported to play a major role in the nature and structure of ongoing partnerships forged between the CGH and its institutional collaborators.

In terms of Equity, this study reports that existing partnering institutions and research bodies with whom the CGH works, feel the need *for inclusion, adequate sharing of resources, and respect for different capacities* amongst others, as seen in Fig. 1. Equity in health research remains a constructive core concept and element for achieving the goals and objectives of the established partnership. Partnerships that are based on mutual respect and openness to learn from each other are central to effective capacity building for research [8]. Therefore for research partnerships in global health to become successful, equity remains essential as a key part of the established partnership.

This study shows that who gets what, when, how, and why is an important component. Therefore the benefit derived from global health partnership is instrumental to its formation. As a core concept, the benefit individuals and institutions derive from research partnerships in global health can be centred on certain attributes such as *having a rewarding experience, knowledge exchange, mutually beneficial* and *skills generation* as seen in Fig. 1. Airhihenbuwa et al. [8] reports that as result of their focus on capacity building, the project provided students in Southern African universities the opportunity to develop their research skills to tackle HIV and AIDs in their country. Knowing the benefit attached with specific projects remains a fundamental indicator which determines the success of research partnerships in global health.

“Effective communication is critical to the achievement of mutual goals, an understanding and prudent use of proven communication principles is a sine qua non for success” [35]. This study reports that communication is crucial for research partnerships to succeed. Having a transparent, open, honest and unambiguous communication strategy laid out before the commencement of research partnerships is vital; that is communications such as meeting notices being circulated on time, agendas, and requests for reports being kept real and simple [35]. In addition, the ability of partners to seek clarification meant that such communication channels were open to all and remained effective, as seen in this study.

Two additional findings—Resolution and Leadership—that are not fully interrogated in existing literature (with the exception of CCGHR's “Partnership Assessment Tool which considered resolution in some depth), emerged as core concepts in our study. Key issues were raised regarding the potential dissolution of partnerships, suggesting that a) resolution is central to ensuring that research partnerships remain successful; b) collaborating partners should have the foresight to agree on strategies that will minimise conflicts or challenges that may arise over the course of the partnership; and c) formal MOUs between partners need to have pre-defined criteria and consider appropriate exit strategies. Our results emphasized the need for good management of operations and relationships underlying all processes of successful partnerships. Such skill sets, while often underplayed, are advantageous in the contextualisation and application of core concepts towards outcomes and goals.

Partnerships, either negative or positive, are often influenced by contextual factors and positionality of the partners themselves. Supported by the literature and our data, these dynamics can be reflective of underlying power imbalances and persisting inequalities, for instance between north—south, public-private, or global—local collaborations. We believe that disparity of capacities, particularly between North-South partners, needs to be reflected in order for partnerships to be equitable.

## Conclusions

We suggest that partnerships, particularly large and complex ones, can function well if *all* partners engage with the seven core concepts presented in this framework.

While each partnership needs to be contextualised and adapted to the *relational* and *operational* aspects involved, the framework presented here can be used to inform and guide the process of successful partnership development. The CGH intends to use the framework to guide the development of new partnerships and improve existing partnership practice.

The next phase of this process will be to disseminate these results to our participants and, using a Delphi technique, develop indicators that can be used to validate the framework.

## Appendix

**Table 1:** Selected sample of CGH partnerships (2005—present)

South/North	Collaborating Institution/Organisation	Location	Partnership/Project Description
North/North			
S/N	UNESCO and UNESCAP	Malaysian, Timor L’este, Cambodia	*Inclusive Asia*: Policy revision and development
S/N	National University of Malaysia	Malaysia	Doctoral Research (Indigo)
N/N	University College Dublin	Ireland	
S/N	Addis Ababa University, Hamlin Fistula Hospital	Ethiopia	Doctoral Research (Indigo)
S/N	University of Malawi	Malawi	Doctoral Research (Indigo)
S/N	University of Ibadan, Nigeria	Nigeria	Doctoral Research (Indigo)
S/N	Makerere University	Uganda	Doctoral Research (Indigo)
N/N	Columbia University	USA	Doctoral Research (Indigo)
N/N	Harvard Medical School	USA	Doctoral Research (Indigo)
N/N	Cochrane Centre, Evidence Aid	UK	Doctoral Research (Indigo)
N/N	Ulster University	UK	
S/N	World Health Organization	Global	
S/N	World Vision	Sierra Leone, Uganda, Kenya, Tanzania, Mauritania	Access: Infant and Maternal Health (AIM-Health)
S/N	Afhad University for Women,	Sudan, Malawi,	*EquitAble*: Policy revision and development
S/N	University of Malawi, Ministry of Person with Disability	Malawi	Equitable
S/N	Human Sciences Research Council,	South Africa	Equitable
S/N	University of Stellenbosch	South Africa	Equitable
S/N	Norwegian Institute of Technology	Norway	Equitable
S/N	University of Namibia	Namibia	Equitable
S/N	Ministry of Health	Mozambique	
S/N	National Institute for Medical Research	Tanzania	
S/N	Ifakara Health Institute	Tanzania,	Support, Train, and Empower Managers (STEM)
S/N	Eduardo Mondlane University	Mozambique	Support, Train, and Empower Managers (STEM)
S/N	Kenya Medical Research Institute	Kenya	
N/N	UNICEF	USA	
N/N	Johns Hopkins University	USA	

## Additional file

Additional file 1: Questionnaire administered during the consultative process with partners (Phase 1), includes six open-ended questions about important features of successful partnerships. (DOCX 32 kb)
