# Supplementary material for: Developing a framework for successful research partnerships in global health
Source: Global Health. 2016 May 6;12:17. doi: 10.1186/s12992-016-0152-1 (PMC4859962; doi:10.1186/s12992-016-0152-1)
Supplement: Additional file 1: — Questionnaire administered during the consultative process with partners (Phase 1), includes six open-ended questions about important features of successful partnerships. (DOCX 32 kb) [file 12992_2016_152_MOESM1_ESM.docx]

Appendix 2: Questionnaire

**Towards Successful Global Health Research Partnerships**

Partners differ in their location, type of organisation, and many other factors. In order to make these partnerships as effective as possible we want to understand just what our partners consider – *from their own perspective* - to be the characteristics of successful research partnerships.

To this end, we invite you to share some thoughts with us. This will only take you 5 minutes and we would be really grateful for your time and thoughts on it.

1. Please name some of partnerships you have been involved in with us and with others?
2. Please try and identify between 5 and 10 features that you feel are important for good partnerships and, if necessary, provide a brief description of why.

| Number | Feature | Description |
| --- | --- | --- |
|  |  |  |
|  |  |  |
|  |  |  |
|  |  |  |
|  |  |  |
|  |  |  |
|  |  |  |
|  |  |  |
|  |  |  |
|  |  |  |

1. In your opinion how best are partnerships created?
2. In your opinion what are the critical factors for making partnerships fair?
3. What do you think is the value of partnership?

Thank you for spending the time to answer these questions.

Having gathered this information from a broad range of partners we will summarise the features identified. May we share the results of this with you?
